# Superhydrophobic and highly moisture-resistant PVA@EC composite membrane for air purification†

**DOI:** 10.1039/d2ra05798k

**Published:** 2022-12-07

**Authors:** Zhiqian Liu, Linli Qin, Sijia Liu, Jing Zhang, Junhua Wu, Xinquan Liang

**Affiliations:** School of Light Industry and Food Engineering, Guangxi University Nanning 530000 Guangxi P. R. China 19890011@gxu.edu.cn; Guangxi Academy of Sciences Nanning 530000 P. R. China xxwjh@sina.com

## Abstract

Electrospun fiber membranes have great potential in the field of air filtration because of their high porosity and small pore size. Conventional air filtration membranes are hydrophilic, leading to weak moisture-barrier properties, which hinders their application in high-humidity environments. In this study, eugenol was added to polyvinyl alcohol and ethyl cellulose (EC) for electrospinning and electrospraying, respectively, of superhydrophobic bilayer composite fiber membranes to efficiently filter particulate matter (PM) in air. Owing to its surface microstructure, electrosprayed EC increased the water contact angle of the PVA membrane from 142.8 to 151.1°. More importantly, the composite air-filter membrane showed a low filtration pressure drop (168.1 Pa) and exhibited high filtration efficiencies of 99.74 and 99.77% for PM_1.0_ and PM_2.5_, respectively, and their respective quality factors were 0.0351 and 0.0358 Pa^−1^. At the same time, the filtration performance of the air filtration membrane remained above 99% at high air humidity. This work reports composite membranes that can effectively capture PM of various sizes and thus may provide a reference for the manufacturing of green air filters for high-humidity environments.

## Abstract 2

Electrospun fiber membranes have great potential in the field of air filtration because of their high porosity and small pore size.

## Graphical abstract

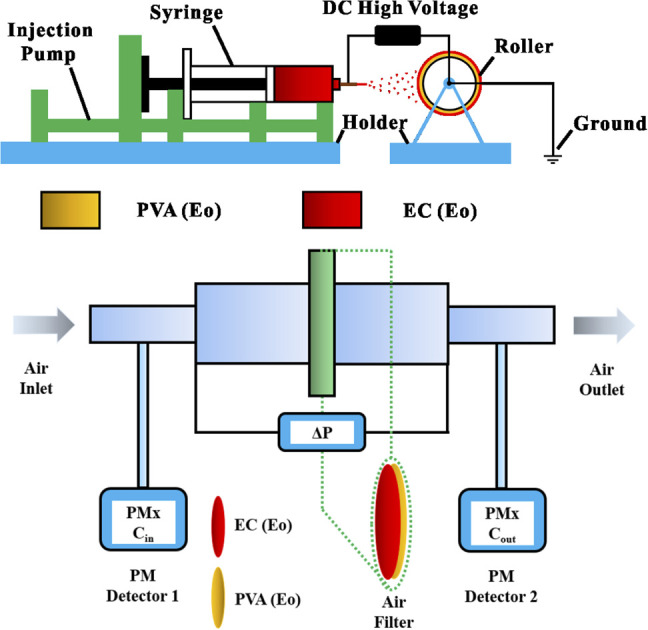

## 1 Introduction

Over the past few decades, air pollution has become a public health hazard owing to various industrial activities, such as waste incineration, and release of production exhaust and vehicle exhaust gases.^1^ Particulate matter (PM) is a pervasive air pollutant that causes serious health problems. PM is classified into PM_0.3_, PM_0.5_, PM_1.0_, PM_2.5_, and PM_5.0_, representing particle sizes below 0.3, 0.5, 1.0, 2.5, and 5.0 μm, respectively.^2^ These particles pose a serious threat to human health and contribute to many air-pollution-related diseases and consequently higher mortality.^3^ In addition to eliminating pollutant emissions at the source, air filtration is an important method for reducing air pollution at a low cost.^4,5^ In recent years, the use of air filtration materials has dramatically increased worldwide owing to the widespread concern regarding PM pollution.^6^ Conventional wearable devices, air filtration membranes, and air filters currently rely on mechanisms, such as Brownian diffusion, direct interception, inertial impact, and gravitational settling, to effectively block large particles.^7^ Ideally, air-filter membranes should have high air flux, low resistance, and high PM_*x*_ filtration efficiency.^8^ For this purpose, various materials and techniques have been developed: electrospraying,^9^ solution blow-spinning,^3^ electrospinning,^10^ and metal–organic framework-based membranes.^5^ The use of nanofiber composites as air-filter membranes is an effective solution for filtering PM_*x*_.^11^ Various air-filter membranes based on nanomaterials or polymers with high dipole moments have been developed because electrospun nanofibers increase the possibility of particle deposition on the fiber surface owing to their small diameter and high specific surface area.^12,13^

Among electrospun materials, polyvinyl alcohol (PVA) is a green, non-toxic, and degradable linear polymer that can be used in filtration membranes, wound dressings, *etc.*^10,14,15^ Zhao *et al.*^16^ used cellulose nanofibrils, PVA, and bamboo-activated carbon to construct a hybrid freeze-drying dual air filtration system, the PM_2.5_ filtration efficiency of which reached 99.69%. Zhang *et al.*^17^ prepared a PVA/cellulose nanocrystal-electrospun nanofibrous air filter for PM removal and reported a removal efficiency above 95%. This shows that the PVA-based composite air-filter membranes have a high PM removal efficiency and a unique advantage as an air-filter membrane carrier material. However, some air filtration membranes made of hydrophilic materials exhibit severe degradation under high air humidity, thus limiting their application in high-humidity environments.^18^ Hydrophobic-modified materials with water contact angles (WCAs) greater than 90° have received considerable attention because of their potentially broad application scope. Superhydrophobic surfaces exhibit low water adhesion and excellent non-wetting behavior, forcing water droplets to form beads, thereby protecting the surface structure of the membrane.^19^ Liu *et al.*^20^ reported a methyltrimethoxysilane super-hydrophobic-modified cellulose nanofiber aerogel for the efficient filtration of PM in air, with removal efficiencies of 99.31 and 99.75% for PM_1.0_ and PM_2.5_, respectively. Therefore, enhancing hydrophobicity is a viable method to enhance the moisture-proof filtration performance of the filter membrane.

Ethyl cellulose (EC) is an etherification-modified hydrophobic cellulose that is widely used as a binder, filler, and coating in cosmetics, food, and pharmaceuticals.^21^ Its main advantages include low surface energy, low cost, non-toxicity, and biocompatibility.^22^ Electrospraying is a variant of electrospinning, where the morphology of the spinning solution of dissolved polymers in a high-voltage electrostatic field jet is affected by the molecular weight and concentration of the polymer,^23^ whereas the molecular weight of EC determines the presentation of electrostatic sprays rather than that of electrospinning.^24^ Liu *et al.*^25^ prepared an EC/gelatin-electrospun composite film loaded with zinc oxide (ZnO) nanoparticles and reported that their interaction as a filler with the polymer increased the WCA of the EC composite film from 119.3 to 134.0°. Although the hydrophobicity of EC can be enhanced by adding hydrophobic substances to it, reaching the super-hydrophobic level,^26,27^ which is important for enhancing the moisture resistance and filtration efficiency of the composite membrane, requires the modulation of its surface microstructure, for example, through electrospraying.^24^ Liu *et al.*^28^ found that the volatilization rate of the solvent during electrospraying changes the surface morphology and microstructure of the formed particles. Eugenol (Eo), a hydrophobic aromatic compound found in natural essential oils,^29^ is used as a food additive, drug, and component in cosmetics.^30^ Owing to the volatility of Eo, we hypothesized that the addition of Eo would modulate the micro–nanostructure of the electrosprayed EC surface, making the EC particles superhydrophobic, which is in line with the report by Liu *et al.* Thus, Eo, a hydrophobic essential oil, was added to PVA to form an emulsion, which was then electrospun into a fiber membrane. The addition of Eo enhanced the moisture resistance and filtration performance of the composite membrane. To the best of our knowledge, the preparation of superhydrophobic micro–nanostructured particles by EC electrospraying has not yet been reported, and no studies on the use of PVA/EC bilayer composite membranes incorporating Eo for moisture resistance and efficient air filtration have been previously published.

In this study, a superhydrophobic (WCA = 151°) bilayer composite fiber membrane with high PM filtration efficiency was prepared by electrospinning the emulsion of PVA and Eo and electrospraying EC with Eo onto the electrospun membrane. At a low filtration pressure drop (168.1 Pa), the filtration efficiency toward PM_1.0_ and PM_2.5_ was as high as 99.74 and 99.77%, respectively, with respective quality factors (QFs) of 0.0351 and 0.0358 Pa^−1^. The filtration performance of the air filtration membranes remained above 99% under high air humidity. This work reports composite membranes that can effectively filter the PM of various sizes, which is significant for the protection of human health and can provide a reference for the manufacturing of green air filters with high-humidity adaptability.

## 2 Materials and methods

### 2.1 Materials

PVA (*M*_w_ = 1700, 99% alcoholysis) and EC (47% ethyl) were purchased from Shanghai Yuanye Biotechnology Co. Potassium nitrate (KNO_3_, 99.5%), sodium chloride (NaCl, 99.5%), sodium bromide (NaBr, 99.5%), potassium carbonate (K_2_CO_3_, 99.5%), magnesium chloride (MgCl_2_, 99.5%), lithium chloride (LiCl, 98%), and Tween-80 (98%) were purchased from Sinopharm Chemical Reagent Co. Anhydrous ethanol (99.7%) and glacial acetic acid (99.5%) were purchased from Chengdu Kolon Reagent Company (China). Eo (98%) was purchased from Shanghai Aladdin Biochemical Technology Co. All reagents were used without further purification.

### 2.2 Fabrication of electrospun composite membranes

#### 2.2.1 Electrospinning of PVA emulsion

**Fig. 1:**
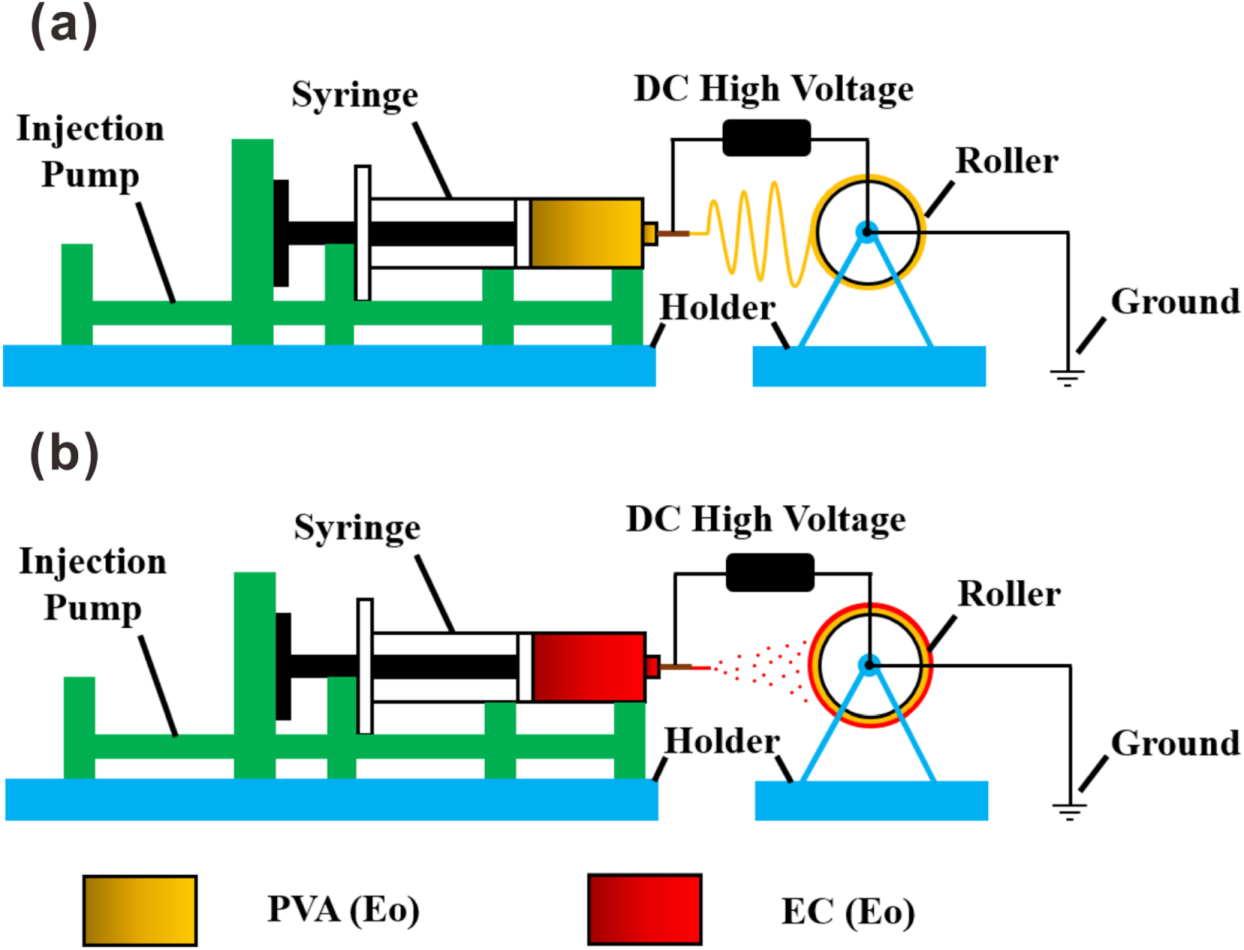
Schematic diagram of the composite air filtration membrane manufacturing process: (a) preparation of PVA(Eo) electrospinning membrane; (b) preparation of EC(Eo) electrostatic spraying.

PVA was dissolved in pure water and heated at 95 °C in a water bath under stirring for 1 h to obtain a 12 wt% solution, which was the pure PVA electrospinning solution. After cooling, for every 100 mL of solution, we added 2 mL of Tween-80 emulsifier and 5 g of Eo dropwise under intense stirring to form the O/W emulsion. The mixture was stirred continuously for 3 h and set aside. The prepared pure PVA electrospinning solution and PVA electrospinning emulsion were loaded into 10 mL medical plastic syringes with 18 G dispensing needles. An electrospinning machine (HZ-02, China) was used to connect the positive electrode of the high-voltage DC power supply. A 20 cm in diameter rotatable drum (grounded) was used as the collection device, and tin foil was wrapped on the surface of the drum as a substrate for collecting the electrospun fiber film. Spinning parameters were the following: distance between the spinneret and the collection device of 15 cm, injection advance rate of 1.0 mL h^−1^, voltage of 15 kV, drum speed of 300 rpm, room temperature (25 °C), and 60–80% ambient humidity. As shown in Fig. 1(a), the film prepared of PVA with added Eo was denoted as PVA(Eo).

#### 2.2.2 EC electrospraying

EC was dissolved in an acetic acid/ethanol (5 : 5, v/v) solution to form a 10 wt% pure EC solution; 5 wt% Eo was added under vigorous stirring, followed by stirring for 3 h. The bilayer film was prepared by electrospraying either EC or EC with Eo onto the PVA(Eo) film as the substrate. As shown in Fig. 1(b), the sprayed layer of EC with Eo was denoted as EC(Eo), and the finished composite film was denoted as PVA(Eo)@EC(Eo). Spinning parameters were the following: distance between the spinneret and collection device of 15 cm, injection advance rate of 1 mL h^−1^, voltage of 20 kV, drum speed of 300 rpm, room temperature (30 °C), and 60–80% ambient humidity.

### 2.3 Characterization of composite film structure and morphology

The surface and cross-sectional fiber morphology structures of PVA, PVA(Eo), EC, and EC(Eo) before and after filtration were observed using scanning electron microscopy (SEM; F16502, The Netherlands). The fiber morphology was analyzed using Nano Measurer v1.2 software: 60 sets of fiber and particle diameter data points were obtained from each SEM image to construct diameter distribution maps. Fourier transform infrared (FT-IR; VERTEX 70, Germany) spectroscopy was performed in a detection wavelength range of 500–4000 cm^−1^ to analyze the chemical composition of the composite film. The specific surface areas of the samples were measured using a TriStar II 3020 automated specific surface area analyzer to perform BET analysis. A KRUSS DSA100 CA meter was used to determine the WCA of the composite membrane surface using the static droplet method with the water droplet volume of 4 μL and precision stainless steel needle tip.

### 2.4 Air filtration and moisture resistance testing

The tests were conducted according to the procedure reported by Fan^31^ and Liu *et al.*^20^ As shown in Fig. 7, the PVA(Eo)@EC(Eo) composite air-filter membrane was fixed on the device, with the EC(Eo) membrane side oriented toward the particle generation device. Non-oily PM particles were generated by burning mosquito coils in a sealed glove box (40 × 40 × 50 cm). A particle counter (DT-9881M, CEM) was used to detect the generated PM particles and control the burning volume to maintain a PM_2.5_ concentration of approximately 1000 μg m^−3^. The gas flow rate was controlled at 5.3 cm s^−1^ by an adjustable air pump (YT-712) and rotameter (LZB-6WB), meeting the U.S. Department of Energy test specifications for commercial filters and produced a cylindrical filter with a diameter of 6 cm. PM_*x*_ particles were quantified into five fractions: PM_0.3_, PM_0.5_, PM_1.0_, PM_2.5_, and PM_5.0_. The filtration efficiency was determined using the particle counter (DT-9881M, CEM) by comparing the PM_*x*_ concentration before (*C*_in_) and after (*C*_out_) filtration (*η*, eqn (1)). The pre-filtration pressure (*P*_in_) and post-filtration pressure (*P*_out_) were measured using a pressure drop meter (LK-168, DEYI) connected to both sides of the air purification membrane; the filtration resistance (Δ*P*, eqn (2)) was determined as the difference between these two pressures. (QF, eqn (3)) is commonly used to quantitatively assess the overall filtration performance of composite membranes. A rotameter (LZB-6WB) was used to adjust the air velocity to determine the effect of air velocity on the filtration efficiency and pressure drop.1
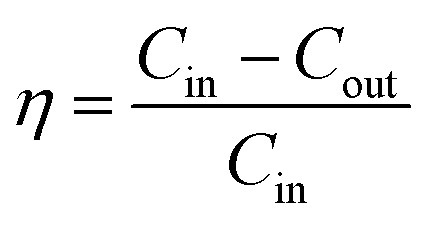

2 Δ*P* = *P*_in_ − *P*_out_

3
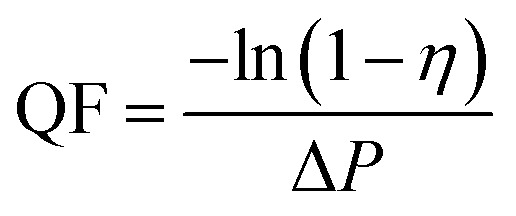


It has been shown that different types of saturated salt solutions can simulate environments with different humidities.^32^ Hong^33^ and Carotenuto *et al.*^34^ used a series of saturated salt solutions: potassium nitrate (90% relative humidity (RH)), sodium chloride (75% RH), sodium bromide (60% RH), potassium carbonate (45% RH), magnesium chloride (30% RH), and lithium chloride (15% RH) to maintain six different relative air humidity levels. Thus, in the present study, a container with saturated potassium nitrate was placed in the chamber where PM_*x*_ was generated to create a stable humidity environment of approximately 90% RH. The filtration performance was tested with reference to a previous method of burning mosquito coils to generate PM_*x*_.

## 3 Results and discussion

### 3.1 Morphology and structure of the composite films

**Fig. 2:**
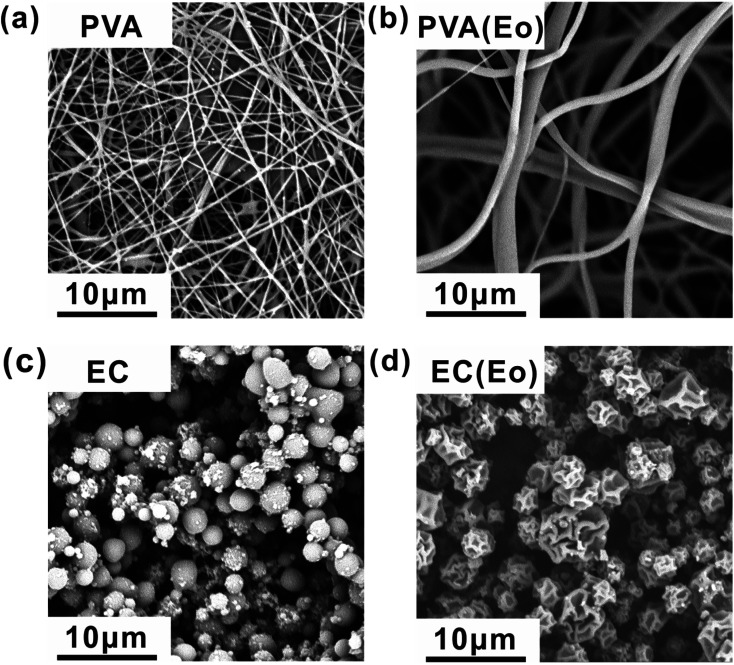
SEM images of each component of the composite air filtration membrane at 8000 magnification: (a) PVA; (b) PVA(Eo); (c) EC; (d) EC(Eo).

SEM micrographs at 8000 magnification (Fig. 2) show the surface morphology of the fibrous films after successful electrospinning and electrospraying. Fig. 2(a) shows the morphology of electrospun film of pure PVA, exhibiting independently dispersed fibers. Notably, the fibers formed by emulsion electrospinning (Fig. 2(b)) have greater dimeters and smoother surface than pure PVA fibers, indicating a more regular fiber morphology of electrospun emulsions of PVA with Eo. Fig. 2(c) presents the surface of the microspheres formed by electrospinning of pure EC, showing their regular spherical shape. By contrast, the electrosprayed EC(Eo) microspheres (Fig. 2(d)) exhibit altered morphology and depressed and wrinkled microsphere surface. According to Mohamed *et al.*,^35^ the rejection of water by solid surfaces largely depends on surface morphology. As mentioned in the Introduction, the rate of solvent evaporation during electrospraying changes the surface morphology and microstructure of the formed particles, resulting in an increased surface area of the EC(Eo) microspheres.^24,28^ Because of the increased contact surface area at the solid–liquid interface, EC(Eo) is expected to have higher hydrophobicity than EC, further validating the hydrophobic effect of Eo-modification of EC.

Cross-sectional and SEM images of the composite membrane before and after the filtration test are provided in ESI Fig. S1.† Fig. S1(a)† presents the boundary morphology of the EC particles and PVA fibers at 3000 magnification, showing that the EC particles are covered with fibers. Fig. S1(b)† shows the morphology of the fibers on the surface of the PVA(Eo) layer after 1 h of continuous filtration of the composite membrane, where PM particles intercepted by the PVA fibers are observed on the fiber surface, indicating that the composite membrane fibers have a certain barrier effect on PM particles.

**Fig. 3:**
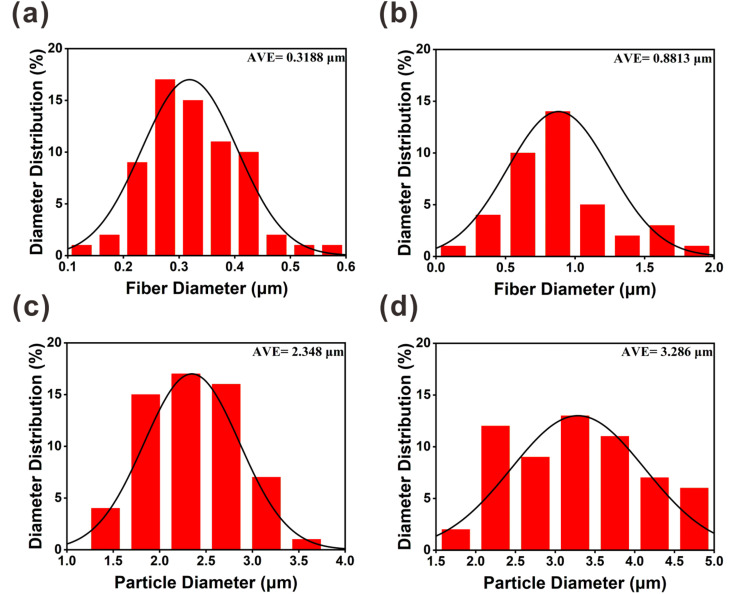
SEM images of the diameter distribution of different components of the composite air filter membrane: (a) PVA; (b) PVA(Eo); (c) EC; (d) EC(Eo).

Fig. 3 shows that the diameter distributions of the electrospun fibers and sprayed microspheres are overall normal. Fig. 3(a) and (b) show that the average fiber diameter of PVA(Eo) (881.3 nm) is greater than that of pure PVA (318.4 nm). As shown in Fig. 3(d), the average diameter of the sprayed microspheres with Eo added was 3.286 μm, which is greater than that of EC without Eo added, as shown in Fig. 3(c). It should be noted, however, that the morphology and diameter of the spun fibers are affected by various process parameters, such as the polymer solution concentration, electric field voltage, applied flow rate, and distance between the drum and needle.^36^

**Fig. 4:**
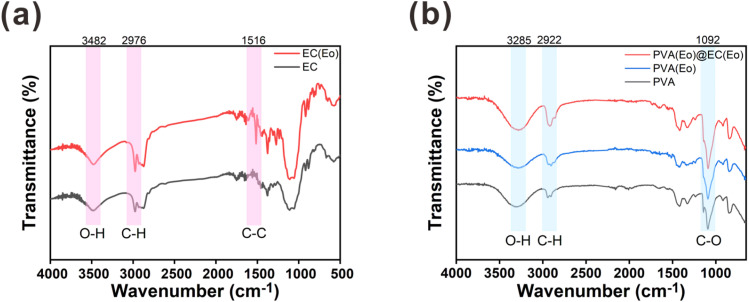
FT-IR analysis of the composite air filter membrane: (a) EC and EC(Eo) characteristic absorption peaks; (b) PVA, PVA(Eo) and PVA(Eo)@EC(Eo) characteristic absorption peaks.

The functional groups on the surfaces of the materials were characterized by FT-IR spectroscopy. As shown in Fig. 4(a), both EC and EC(Eo) exhibit similar absorption peaks.^37^ In particular, the peak at 3482 cm^−1^ was ascribed to the O–H stretching vibration of the alcohol hydroxyl group in EC, and the peak at 2976 cm^−1^ was attributed to the C–H stretching vibration in EC. Notably, the C–C stretching vibration peak of the benzene ring at 1516 cm^−1^ is more pronounced in EC(Eo) than in pure EC, indicating the presence of Eo in EC(Eo).^25^ As shown in Fig. 4(b), PVA(Eo) and PVA(Eo)@EC(Eo) both exhibit the absorption peaks of PVA.^38^ The broad absorption peak at 3285 cm^−1^ was assigned to the O–H stretching vibration in PVA, the absorption peak at 2922 cm^−1^ was ascribed to the C–H stretching vibration, and the absorption peak at 1092 cm^−1^ was attributed to the C–O stretching vibration of the alcoholic hydroxyl group in PVA;^36^ these are the characteristic absorption peaks of pure PVA. PVA(Eo)@EC(Eo) shows a stronger C–O stretching vibration peak of the alcohol hydroxyl group at 1092 cm^−1^ than the pure PVA film, which is attributed to the greater number of C–O groups in EC(Eo). These results are in agreement with those reported by Hosseini,^39^ further validating the successful preparation of the composite film.

We further investigated the microscopic pore structure of the filter by measuring the adsorption and desorption isotherms of N_2_. The results of BET analysis for PVA(Eo) and PVA(Eo)@EC(Eo) adsorbent are shown in Fig. S3(a)†. Shifting in the volume adsorbed for nitrogen adsorption–desorption isotherm of PVA(Eo) nanofiber occurs at lower pressure compared with PVA(Eo)@EC(Eo), which was indicative of a reduction in the pore size. And it can also show a typical type II adsorption isotherm for macroporous structures.^20^ Fig. S3(b)† shows the pore size distribution of PVA(Eo) and PVA(Eo)@EC(Eo) nanofibers. The PVA(Eo)@EC(Eo) has smaller pores compared with PVA(Eo). Based on BJH method,^40^ a narrow pore-size distribution is observed for PVA(Eo)@EC(Eo) nanofiber adsorbent with an average pore size of 3.864 nm and total pore volume of 0.00843 cm^3^ g^−1^. BET analysis showed that the surface area of the PVA(Eo)@EC(Eo) nanofiber was 11.686 m^2^ g^−1^. Also, average pore size and total pore volume of PVA(Eo) nanofiber adsorbent were 12.839 nm and 0.00606 cm^3^ g^−1^. The surface area of the PVA(Eo) nanofiber was 2.470 m^2^ g^−1^. The value of pore diameter of fibers indicated that the surface of PVA(Eo)@EC(Eo) nanofibers was mesoporous. The values of pore size, pore volume and surface area of PVA(Eo)@EC(Eo) are given in Table S1.†

### 3.2 Hydrophobic performance test of composite membranes

**Fig. 5:**
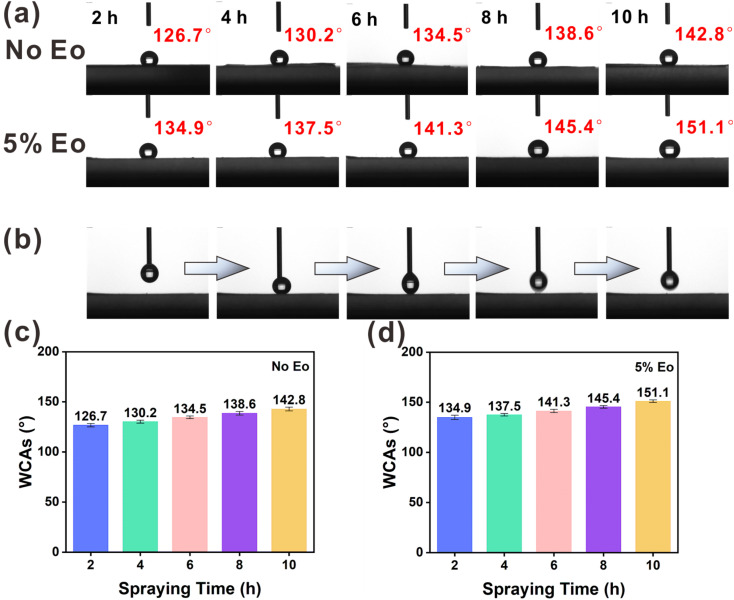
Hydrophobicity analysis of pure EC membrane and EC(Eo) membrane with 5% Eo added on the surface of composite membrane: (a) WCA of EC membrane and EC(Eo) membrane surface; (b) continuous images of water droplet stress contact test for EC(Eo) electrostatic spraying time of 10 h and water droplet volume of 4 μL; (c) WCA histogram of EC membrane surface; (d) WCA of EC(Eo) membrane surface histograms.

In the WCA test, a series of composite membranes were prepared by electrospraying EC and EC(Eo) for 2, 4, 6, 8, and 10 h on top of PVA(Eo) membranes. As shown in Fig. 5(a), (c) and (d), the membrane electrosprayed with pure EC for 2 h exhibits low WCA; however, with the increase in EC film thickness, the hydrophobicity of the film surface substantially increases, with the WCA of the EC film surface reaching 142.8° at 10 h spraying time, suggesting that EC itself has good hydrophobicity. Furthermore, the EC(Eo) shows higher WCA than pure EC films at the same spraying time, indicating that Eo further enhanced the hydrophobicity of the EC films. The WCA of the EC membrane with 5% Eo sprayed for 10 h reaches 151.1°, proving that the EC(Eo) membrane has very good hydrophobicity. As shown in Fig. 5(b), the syringe injection volume was 4 μL, and the droplets were removed by the exiting syringe during the process. No residual water droplets are observed on the surface after the syringe exit, indicating that the surface of the superhydrophobic filter membrane is non-adhesive. Therefore, the membrane with the highest hydrophobicity, EC(Eo) with 5% Eo sprayed for 10 h, was selected as the electrostatic spray coating for the preparation of the composite membranes for subsequent filtration performance tests.

### 3.3 Composite membrane filtration and moisture resistance

#### 3.3.1 Composite membrane filtration performance

**Fig. 6:**
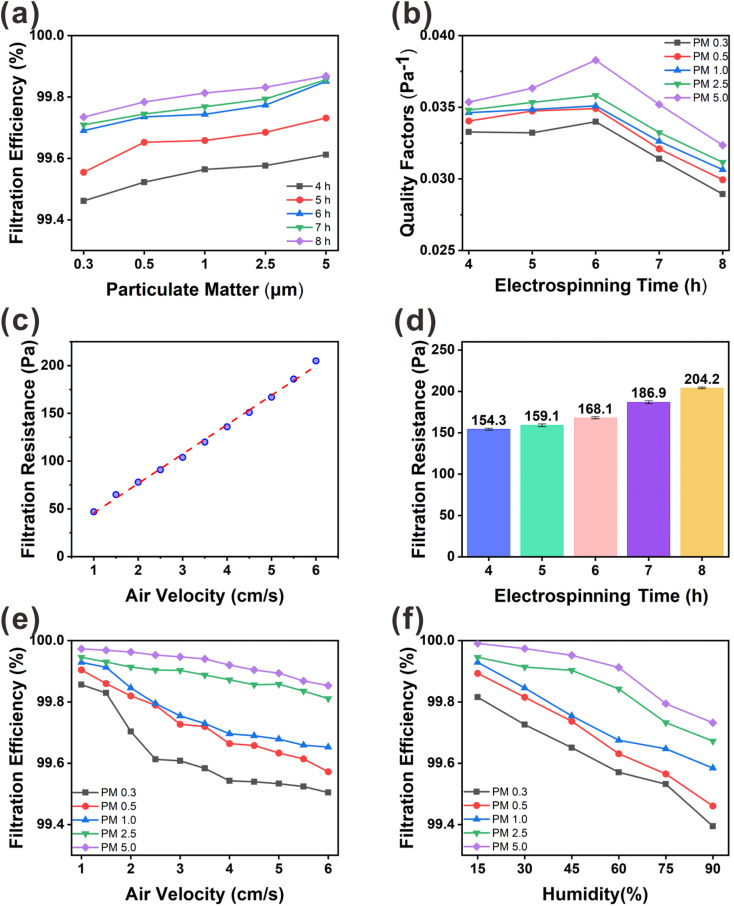
Comprehensive filtration performance of composite air filter membranes for PM_*x*_: (a) filtration efficiency of PM_*x*_; (b) QF factor of PM_*x*_; (c) filtration pressure drop values of the membranes at different wind speeds when the PVA(Eo) spinning time is 6 h; (d) filtration pressure drop values of the membranes at different PVA(Eo) spinning times; (e) filtration efficiency of PM_*x*_ at different wind speeds; (f) filtration efficiency of PM_*x*_ at different humidity.

**Fig. 7:**
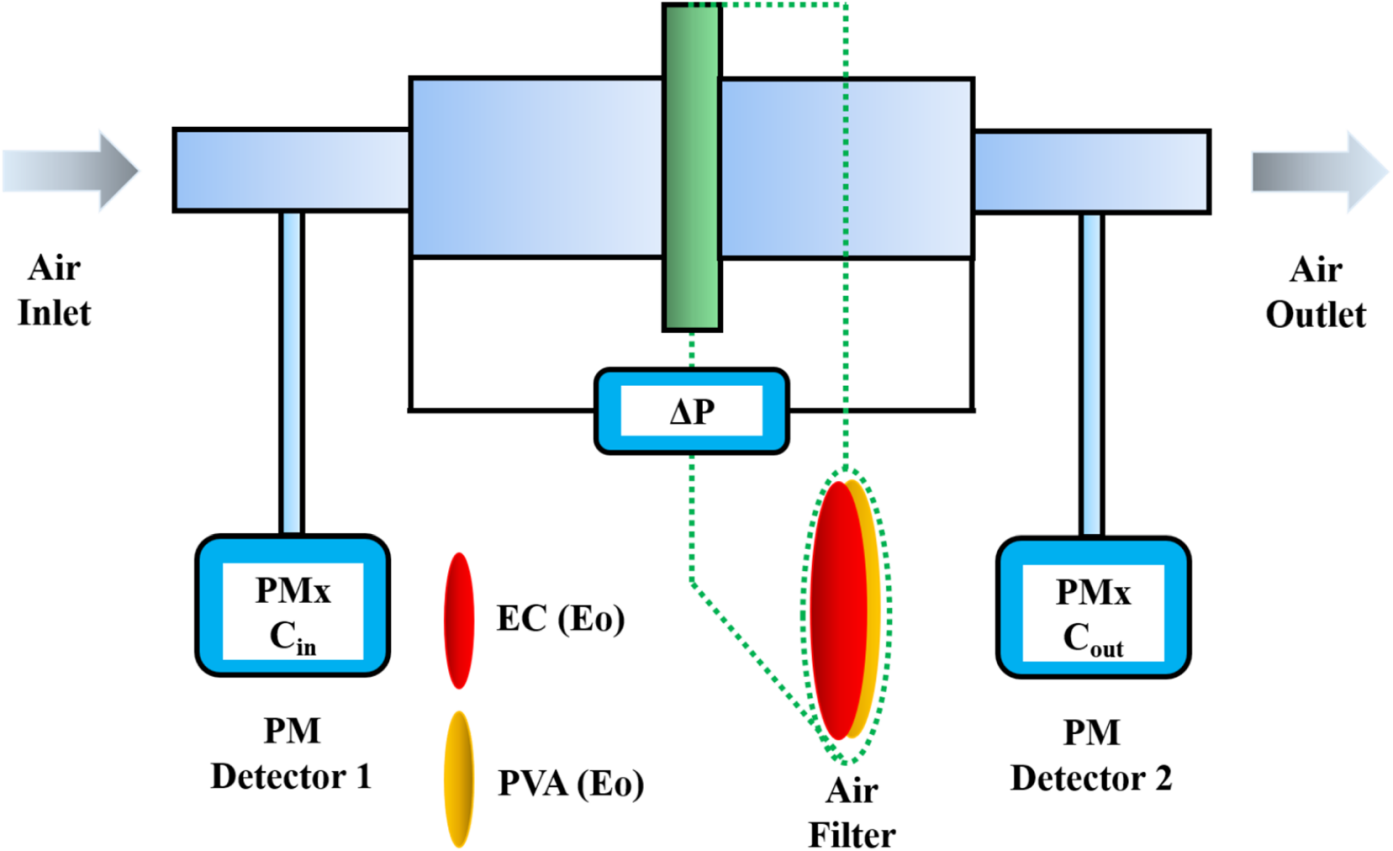
Schematic diagram of air filtration device.

The filtration performance of the composite membrane was tested on simulated non-oily PM_*x*_ produced by burning mosquito coils; the PM_0.3_, PM_0.5_, PM_1.0_, PM_2.5_, and PM_5.0_ filtering performance tests and subsequent measurements were performed at 60% RH. Fig. 7 shows a schematic of the test setup used for the evaluation of the PM_*x*_ filtration performance, with the PM flow rate, pressure drop (Δ*P*), and concentration determined using a commercial detector. As shown in Fig. 6(a), the filtration efficiencies of membranes spun for 8 h reached 99.73, 99.78, 99.81, 99.83, and 99.89% for PM_0.3_, PM_0.5_, PM_1.0_, PM_2.5_, and PM_5.0_, respectively. The filtration efficiency of PM_5.0_ is the highest, and the filtration efficiencies of smaller particles decrease with decreasing particle size. The filtration efficiency increases with the PVA(Eo) spinning time for each particle size. According to the classical filtration theory,^15^ the interception effect dominates at particle sizes larger than 1.0 μm, and inertial deposition, electrostatic adsorption, and Brownian effects occurring at this stage are much weaker. Therefore, the filtration efficiency for small particles (PM_0.3_ and PM_0.5_) is lower, and for large particles (PM_2.5_ and PM_5.0_), the filtration efficiency is higher because the filtration occurs through the combined effect of retention and inertial deposition. In addition, the filtration efficiency of PM_*x*_ increases with PVA(Eo) thickness. The increase in membrane thickness significantly increases the potential for retention, at which point the retention effect was the main influencing factor. Thus, the membrane spun for 8 h exhibited the best filtration performance.

As shown in Fig. 6(d), we measured the filtration pressure drop between the inlet and outlet of the filtration unit for different membrane thicknesses. The lower the filtration pressure drop of the membrane, the higher is the air permeability. With increasing PVA(Eo) spinning time, the path of air permeation through the membrane also increases, resulting in a higher filtration pressure drop. The excessive filtration pressure drop reduced the air permeability, which in turn affected the QF, which is the overall use value of the membrane. At an airflow rate of 5.3 cm s^−1^, the filtration pressure drop was 154.3 Pa for the PVA(Eo) composite membrane spun for 4 h and 168.0 Pa for the composite membrane spun for 6 h; both values are lower than the U.S. Department of Energy standard of 250 Pa for commercial superhydrophobic cellulose nanofiber air-filter membranes with highly efficient filtration and humidity resistance.^20^ As shown in Fig. 6(b), a positive correlation between the QF and PM_*x*_ particle size is observed for the same PVA(Eo) spinning time. However, with increasing PVA(Eo) spinning time, the QF first increases mainly because of the small pressure drop at the membranes spun for 4 and 5 h (in which the QF is primarily affected by the filtration efficiency). With a further increase in film thickness, the filtration pressure drop and the QF decrease. PVA(Eo) spun for 6 h shows the highest filtration quality, with QFs of 0.0340, 0.0349, 0.0351, 0.0358, and 0.0383 for PM_0.3_, PM_0.5_, PM_1.0_, PM_2.5_, and PM_5.0_, respectively. The filtration efficiencies of the composite membrane with PVA(Eo) spun for 6 h for PM_0.3_, PM_0.5_, PM_1.0_, PM_2.5_, and PM_5.0_ are 99.69, 99.73, 99.74, 99.77, and 99.85%, respectively. Thus, the PVA(Eo) membrane with a spinning time of 6 h was optimal for subsequent experiments. To investigate the significance of Eo incorporation into PVA, we also compared the filtration performance of PVA(Eo)@EC(Eo) and PVA@EC(Eo) composite membranes (Fig. S2†) and found that the filtration performance of the latter membrane is overall lower than that of the former, with the differences in PM_0.3_ and PM_0.5_ filtration efficiencies being particularly pronounced. These results were attributed to the release of Eo from the PVA(Eo) fibers to the membrane surface as filtration proceeded, increasing the retention of PM particles, especially of PM_0.3_ and PM_0.5_, which are smaller particles that are generally more difficult to retain. This increased the amount of PM retained on the surface of the fibers, which in turn enhanced the overall filtration effect of the composite membrane; this consideration is supported by the surface morphology of the composite membrane after the filtering test (Fig. S1(b)†).

To understand the effect of wind speed on the PVA(Eo)@EC(Eo) composite membrane (6 h PVA(Eo) electrospinning and 10 h EC(Eo) spraying), we tested its filtration efficiency and filtration resistance at different wind speeds. As shown in Fig. 6(c) and (e), the filtration efficiencies of PM_*x*_ are similar to those in Fig. 6(a), that is, larger particles were filtered more efficiently. At wind speeds lower than the conventional 5.3 cm s^−1^, the slower airflow increases the probability of PM being captured by the composite membrane fibers and inertial deposition, leading to higher filtration efficiency. In particular, for PM_2.5_, the filtration efficiency reaches 99.95% at the lowest wind speed. At the same time, with increasing wind speed, the filtration efficiency of the membrane gradually decreases, which is consistent with the results of Liu^20^ and Xu.^32^ For PM_0.3_, PM_0.5_, and PM_1.0_, which are three smallest particle sizes, the filtration efficiency decreases more rapidly than for larger particles. This phenomenon is attributed to the weaker effects of gravity and inertial deposition on the small particles, decreasing their retention on the fibers of the composite membrane with increasing wind speed.^32^ Interestingly, as shown in Fig. 6(c), the filtration pressure drop of the composite membrane shows a strong positive correlation with wind speed. The fitted linear equation for these variables is *y* = (30.78182 ± 0.5804)*x* + (14.99091 ± 2.22905), *R*^2^ = 0.99646, indicating that the filtration pressure drop uniformly increases with wind speed. Particularly, the pressure drop increases from 47.1 to 205.2 Pa as the wind speed increases from 1.0 to 6.0 cm s^−1^.

#### 3.3.2 Moisture resistance of composite membranes

Considering its practical application, the filter membrane should not only have a high filtration efficiency but also excellent humidity resistance. Excessive humidity causes the droplets condensed from water vapor to easily adhere to the composite membrane fibers. The formed droplets collide under the disturbance of airflow and combine into large particles when the collision energy is sufficiently high, thus affecting the filtration efficiency of the composite membrane.^20^ As shown in Fig. 6(f), the filtration efficiency of the composite membrane for PM_*x*_ at 90% RH is lower than that at 60% RH, whereas that at 15% RH is excellent, achieving 99.95% removal of PM_2.5_. The decrease in the filtration efficiency from 99.95 to 99.67% with the increase in RH from 15 to 90% shows that the composite membrane with a superhydrophobic structure is also affected by humidity, but the magnitude of the effect is acceptable. This proves that the PVA(Eo)@EC(Eo) composite membrane is effective in humid environments.

## 4 Conclusion

In this study, superhydrophobic composite membranes with high filtration performance and high moisture resistance were prepared by electrospinning the emulsion of PVA and Eo, followed by the electrospraying of EC with Eo onto the surface of electrospun membranes. The results showed that the addition of Eo to PVA and EC increased the hydrophobicity and filtration efficiency of the composite membranes. The highest WCA of 151.1° was observed in the PVA(Eo)@EC(Eo) composite membrane with 5 wt% Eo, prepared *via* PVA(Eo) electrospinning for 6 h and EC(Eo) electrospraying for 10 h. In terms of filtration performance, at the standard air velocity of 5.3 cm s^−1^ and 60% RH, the filtration efficiency for PM_0.3_, PM_0.5_, PM_1.0_, PM_2.5_, and PM_5.0_ reached 99.69, 99.73, 99.74, 99.77, and 99.85%, respectively. A relatively low Δ*P* (168.1 Pa) effectively increased the QF of the membrane, and owing to high humidity resistance, the PM_2.5_ filtration efficiency reached 99.67% at 90% RH. This indicates that the prepared PVA(Eo)@EC(Eo) composite membrane possesses excellent moisture resistance and high filtration performance, thus making it promising for applications in air purification, medical masks, and industrial waste gas treatment.

## Abbreviations

PVA: Polyvinyl alcohol
EC: Ethyl cellulose
Eo: Eugenol
QF: Quality factor
WCA: Water contact angle
SEM: Scanning electron microscope
PM: Particulate matter
FT-IR: Fourier transform infrared
RH: Relative humidity
NaBr: Sodium bromide
KNO_3_: Potassium nitrate
NaCl: Sodium chloride
K_2_CO_3_: Sodium carbonate
MgCl_2_: Magnesium chloride
LiCl: Lithium chloride
BET: Brunner–Emmet–Teller measurements

## Author contributions

Zhiqian Liu: responsible for the design, experiments and drafting of the manuscript; Linli Qin: measurement analysis and auxiliary testing; Sijia Liu: data summarization analysis and discussion; Jing Zhang: auxiliary testing; Xinquan Liang and Junhua Wu: organization, supervision and guidance of the whole work.

## Conflicts of interest

The authors declare no conflict of interest.

## Supplementary Material

RA-012-D2RA05798K-s001
