# Supplementary material for: Superhydrophobic and highly moisture-resistant PVA@EC composite membrane for air purification
Source: RSC Adv. 2022 Dec 7;12(54):34921–30. doi: 10.1039/d2ra05798k (PMC9727828; doi:10.1039/d2ra05798k)
Supplement: RA-012-D2RA05798K-s001 [file RA-012-D2RA05798K-s001.pdf]

Electronic Supplementary Material (ESI) for RSC Advances.
This journal is © The Royal Society of Chemistry 2022

Supplementary data

Superhydrophobic and highly moisture-resistant PVA@EC composite membrane for air purification

Zhiqian Liu[1], Linli Qin[1], Sijia Liu[1], Jing Zhang[1], Junhua Wu[2,*], Xinquan Liang[1,*]

[1] School of Light Industry and Food Engineering, Guangxi University, Guangxi, Nanning 530000, PR China

[2] Guangxi Academy of Sciences, Nanning 530000, China

*Corresponding Email: xxwjh@sina.com (Junhua Wu); 19890011@gxu.edu.cn (Xinquan Liang)

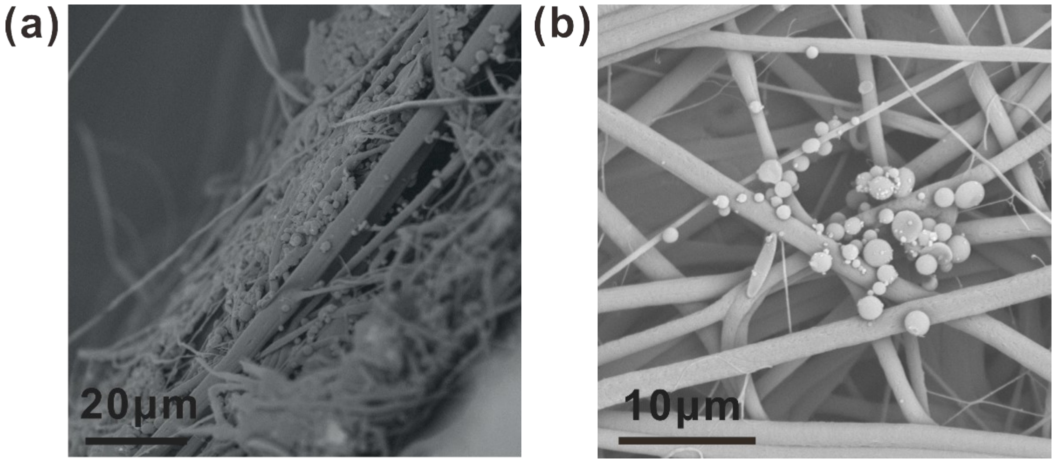

Figure S1. (a) SEM images of cross section of the composite air filtration membrane at 3000 magnification; (b) SEM images of the composite air filtration membrane after filtration at 8000 magnification.

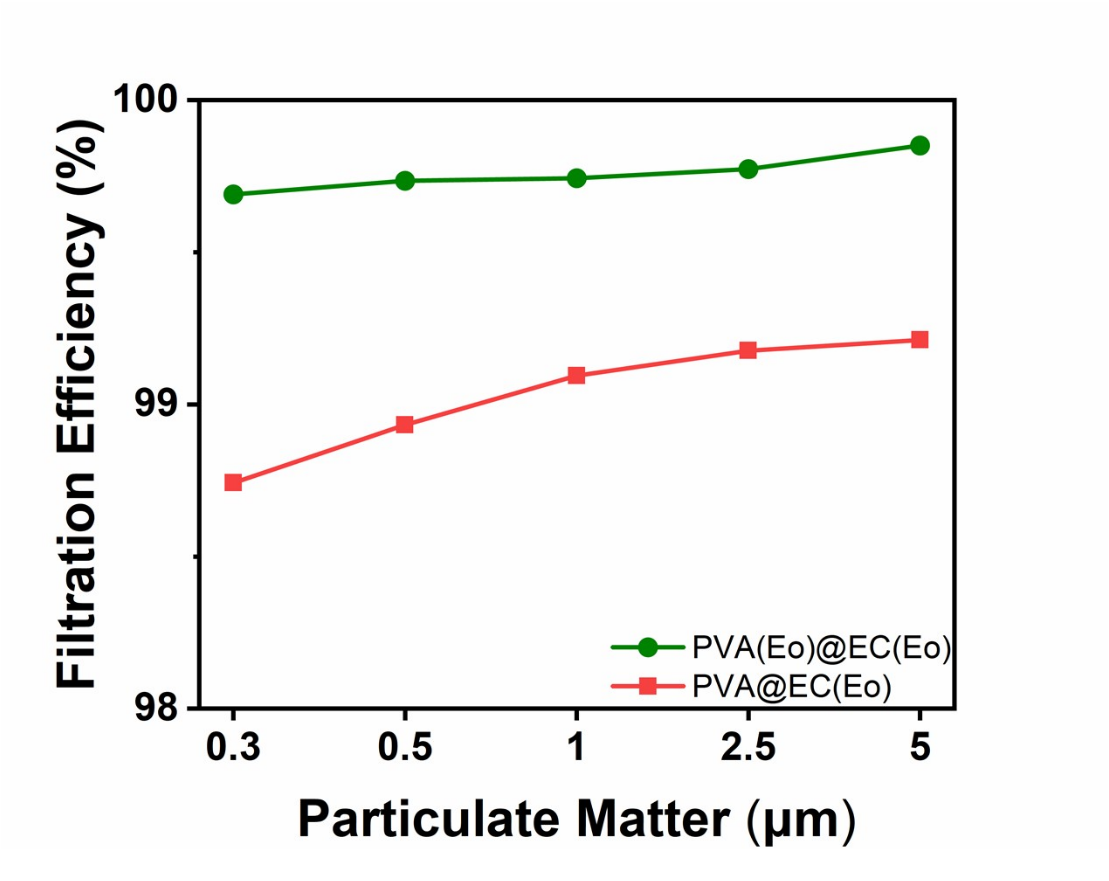

Figure S2. Filtration efficiency of PVA(Eo)@EC(Eo) and PVA@EC(Eo) composite membranes for different particle sizes of PM.

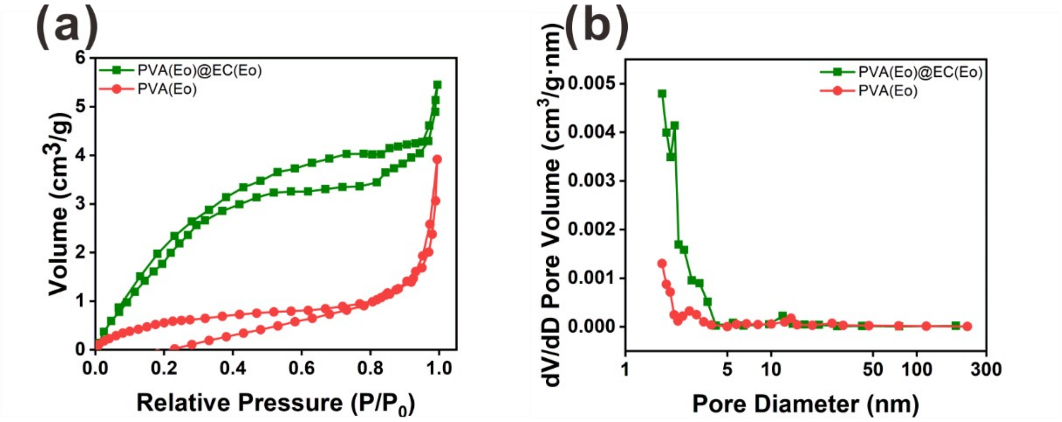

Figure S3. Analysis of the structure and pore size of filters: (a) N_2 adsorption–desorption isothermal line and specific surface area of the filter; (b) Microscopic aperture distribution of filters.

Sample	Surface area (m^2/g)	Pore volume (cm^3/g)	Pore diameter (nm)
PVA(Eo)@EC(Eo)	11.6860	0.008426	3.8640
PVA(Eo)	2.4700	0.006061	12.8392

Table S1. Physical properties of PVA(Eo)@EC(Eo) and PVA(Eo) nanofibers.
